# Low back pain and associated risk factors among medical students in Bangladesh: a cross-sectional study

**DOI:** 10.12688/f1000research.55151.2

**Published:** 2022-02-08

**Authors:** Shabbir Ahmed Sany, Taukir Tanjim, Md Ikbal Hossain

**Affiliations:** 1 Department of Community Medicine, Faridpur Medical College, Faridpur, Dhaka, Bangladesh; 2 Project Research Physician, International Centre for Diarrhoeal Disease Research, Dhaka, Bangladesh

**Keywords:** Bangladesh, Low back pain, Medical students, Prevalence, Risk factors.

## Abstract

**Background:** Low back pain (LBP) is one of the leading causes of disability worldwide. Different studies showed the high prevalence of LBP among medical students. However, no study has been conducted on Bangladeshi medical students to estimate the prevalence of LBP. This study determined the prevalence, characteristics, and associated risk factors of LBP among medical students in Bangladesh.

**Methods:** A cross-sectional study was conducted from October to December 2020 among randomly selected 270 medical students and medical interns in Faridpur Medical College, Bangladesh, using an online questionnaire. In data analysis, chi-square test and binary logistic regression were performed, and a p-value of < 0.05 was regarded as statistically significant.

**Results:** A total of 207 participants responded fully to the survey, and were included in the analysis. The mean age of the participants was 22.4 ± 1.9 years. The point, 6-month, and 12-month prevalence of LBP was 25.6%, 46.9%, and 63.3%, respectively. In most participants, LBP was localized (53.2%), recurrent (64.9%), non-specific (70.8%), affected for a short period (55%), and relieved without receiving any treatment (60.4%). Participants who had a significantly higher 12-month prevalence of LBP included females (72.2% vs 52.2%), with BMI >25 kg/m ^2^ (73.2% vs 56.7%), those who performed physical activity at low to moderate frequency (72.4% vs 29.5%), those who spent > 6 hours/day by sitting (71.3% vs 45.3%), and those who did not have enough rest time (92.7% vs 56%). Ergonomic features of chairs, such as having back support, adjustable back support, and adjustable sitting surface, significantly (p < 0.05) influenced the outcomes.

**Conclusion:** The prevalence of LBP among medical students in Bangladesh was high, and most of the risk factors associated with the high prevalence of LBP were modifiable. Hence, LBP can be prevented by implementing preventive strategies and providing ergonomic training and physical activity facilities.

## Introduction

Low back pain (LBP) is considered the single leading cause of disability-related musculoskeletal conditions globally. ^1^^–^^3^ Researchers showed that 70-80% of people suffer from LBP at least once in their lifetime, ^2^^,^^4^ and 18% of the people suffer from LBP at any given time. ^5^ The healthcare-related costs due to LBP are increasing; hence it is becoming a burden in developed countries, as well as in low-income and middle-income countries. ^3^ In Bangladesh, the prevalence of LBP ranges between 18.6-60.8%. ^6^^–^^9^

Individuals of all ages, including young people and students, can be affected by LBP. ^10^^–^^13^ Medical students are at high risk of developing LBP as they have highly demanding curricula that facilitate a sedentary lifestyle, stressful routines, fewer sleeping hours, long hours of study, hospital training, and classes. ^5^^,^^13^^,^^14^ It is therefore essential to identify the potential risk factors that lead to LBP at an early phase of their career. Prolonged exposure to these risk factors increases wear and tear of the back and consequently raises the injury rate in older age that leads to recurrent and chronic LBP. ^15^^–^^17^ Moreover, Burton *et al*. ^18^ addressed the modification of risk factors as the most crucial prevention strategy of LBP. However, very few studies ^5^^,^^10^^,^^14^^,^^19^^–^^24^ have been conducted that evaluated the prevalence and potential risk factors associated with LBP among medical students. These studies showed a high prevalence of LBP. However, regarding the associated risk factors, the findings were inconsistent.

In Bangladesh, every year, approximately 10,500 students are admitted to 37 public, 70 private, and six armed forces medical colleges. ^25^ To achieve an MBBS (Bachelor of Medicine and Surgery) degree in Bangladesh, students must study for at least five years, and after graduation, they have to complete a compulsory one-year training at the medical college hospital as medical interns. Hence, more than 50,000 students are studying MBBS courses in Bangladesh at any given time. Despite the high number of this specific vulnerable population, no study has been conducted to evaluate LBP prevalence among Bangladeshi medical students. Mondal *et al.* reported that three in every five physiotherapists in Bangladesh suffer from LBP. ^7^ That showed that the people involved in the health sector are at higher risk of developing LBP. Hence, it is essential to conduct more studies to determine the prevalence and risk factors of LBP among health science students and health professionals in Bangladesh so that the results can be compared to know the exposure better, which will help to take necessary initiatives to lessen its impact. Therefore, we aimed to conduct this study to determine the prevalence of LBP and its characteristics and identify the risk factors associated with LBP among medical students of a typical public medical college in Bangladesh.

## Methods

### Study settings and population

This cross-sectional study was conducted on MBBS students (first year to final year) and medical interns in Faridpur Medical College from October to December 2020. Every year, around 120 students are admitted to this medical college; hence typically, there are about 600 students and 120 medical interns in the medical college at any given time.

The study’s sample size was calculated as 251 using OpenEpi version 3.1, assuming 47.5% as the estimated prevalence rate ^10^ at a 95% confidence level with 5% precision. The sample size was calculated based on an Indian study ^10^ as the Bangladeshi medical students have relatively similar curriculum, clinical class exposure, study load, and social and cultural demographics as Indian medical students. Because of the possibility of sample loss, the final sample size was determined as 270. Forty-five students from each batch (1 ^st^ year to 5 ^th^ year) and 45 medical interns were selected randomly by lottery method using their roll number. The study was reported following the STROBE guidelines for reporting observational studies. ^26^

*Inclusion and exclusion criteria*

Full-time medical students studying 1 ^st^ year to 5 ^th^ year at Faridpur Medical College and medical interns were included in that study. Students who refused to give full consent to participate in the study were excluded.

### Instruments

An online, standardized, self-administrated questionnaire ^27^ was used for data collection. The questionnaire was in English language and had three sections. Different sections of the questionnaire were adapted from the minimal dataset reported by Deyo *et al.*, ^28^ and the questionnaires that were validated and used in previous studies. ^29^^,^^30^

Section 1 contained five questions related to socio-demographic data, including gender, age, height, weight, and educational level. Body mass index (BMI) was calculated as weight (in kg) divided by height squared (in meters). In Section 2, lifestyle-related questions, such as exercise frequency, smoking habits, total sitting time in a day (in hours), type of activity mostly done in a day, and availability of enough rest time, were inquired. In addition, the ergonomic characteristics (availability of back support, adjustable back support, adjustable sitting surface) of participants’ chairs were also assessed in that section. To determine the prevalence of LBP at different time points, participants were asked whether they suffered from LBP during the survey, the last 6 months, and the last 12 months (dichotomous scale, Yes/No) in Section 3. This section also included data regarding the first appearance, causes, and aggravating factors of LBP; duration and episode of LBP in the last 12 months; the presence of associated leg pain; and type of received treatment.

The questionnaire was piloted on 15 students before administration in the study to confirm the appropriateness and understandability of questions. The questionnaire was modified according to the feedback, and the responses from the pilot study were not included in the main study.

### Operational definitions and study variables

• Point prevalence: Presence of LBP at the time of the survey. ^5^
• 6-month prevalence: Had at least one episode of LBP in the last 6 months. ^5^
• 12-month prevalence: Had at least one episode of LBP in the last 12 months. ^5^

Dependent variable:

• Low back pain (LBP): LBP is the pain, muscle tension, or stiffness localized below the costal margin and above the inferior gluteal folds with or without leg pain. ^31^

Independent variables:

• Body mass index (BMI): the weight in kilograms, divided by height in meters squared. ^32^
• Aggravating factors of LBP: The activities that cause the low back symptoms to recur.
• Exercise: A controlled, structured, and repetitive subset of physical activity with an ultimate or intermediate objective to improve or maintain physical fitness. ^33^

### Data collection

We used the online survey software from Google Drive to conduct the survey and record the responses. The weblink of the questionnaire was sent to selected participants via email with a cover letter that informed the objective of the study and assurance confidentiality of the responses. Participants’ full consent was taken before collecting the data, and they had the right to withdraw anytime without completing the questionnaire. We did not offer any incentives or rewards for participation.

### Data analysis

After receiving responses from the participants, the accuracy and completeness were checked manually, and data were cleaned when required. All statistical analyses were performed using IBM Statistical Package for Social Sciences (SPSS) Version 26. Descriptive statistics were calculated, and the continuous variables were summarized as mean and standard deviation, whereas the categorical variables were summarized as frequency and percentage. Bivariate analysis using the chi-square test was performed to evaluate the variables associated with LBP at different time points. In addition, binary logistic regression was applied to determine the relative odds of occurrence of LBP in the last 12 months due to the presence of a particular factor. The results were presented with an adjusted odds ratio (OR) and confidence intervals for 95% (95% CI). All statistical analysis was set at a 5% level of significance (p < 0.05).

### Ethics

Permission was taken from the Ethical Review Committee (ERC) of Faridpur Medical College.

## Results

### Characteristics of participants

**Figure 1.**
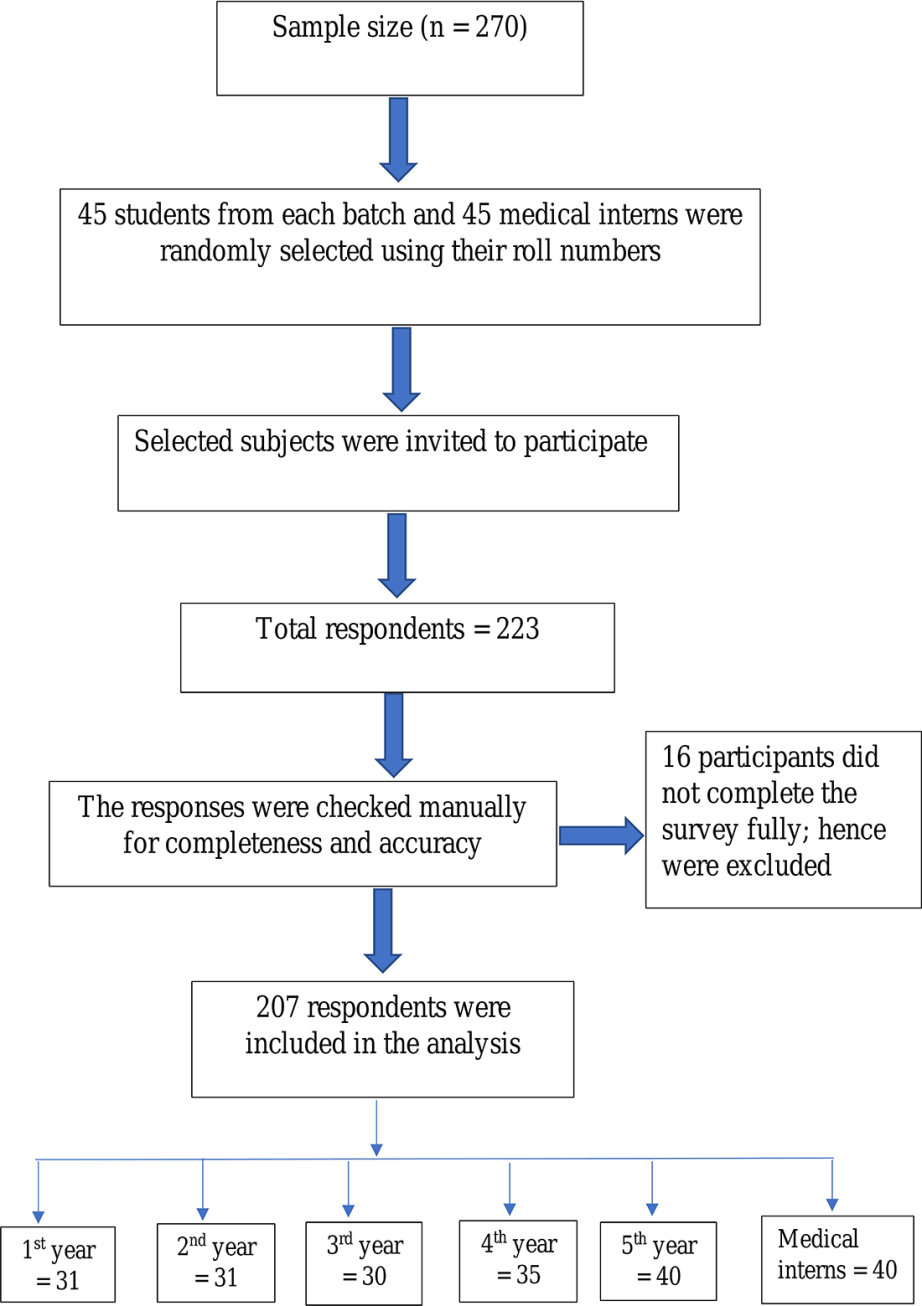
Flow chart of the participants’ inclusion for analysis.

A total of 223 subjects responded to the survey, with a response of 82.6%. However, 16 participants did not complete the survey fully; hence they were excluded. Eventually, 167 medical students and 40 medical interns participated entirely in the study and were included in the analysis. Breakdown of the students was: 31 (15%) in the first, 31 (15%) in the second, 30 (14.5%) in the third, 35 (16.9%) in the fourth, and 40 (19.3%) in the final year of MBBS course ( Figure 1).

Among all participants, 44.4% were males, and 55.6% were females. The mean age of the participants was 22.4 ± 1.9 years, ranging between 19 and 27 years. Based on the mean age, participants were divided into ≤ 21, 22 – 24, and ≥ 25 age groups. Regarding weight, participants were divided into two groups, namely, below normal to normal weight (BMI ≤ 25 kg/m ^2^) and above normal weight (BMI > 25 kg/m ^2^). Three out of five participants (61.4%) had BMI ≤ 25 kg/m ^2^.

The frequency of physical activity was categorized into three groups: Low level (< 4 times/month), moderate level (1-4 times/week), and high level (≥ 5 times/week). Among all participants, almost half (45.4%) performed a low level of physical activity, the majority (83.1%) were non-smokers, nearly half (48.3%) reported that they performed most of their daily activities by sitting, more than two-thirds (69.1%) of participants spent ≥ 6 hours/day in sitting, and four out of five participants (80.2%) had enough rest time. Moreover, the majority (76.3%) used chairs with back support, almost two-thirds (65.2%) used chairs with nonadjustable back support, and more than half (55.6%) used chairs without an adjustable sitting surface ( Table 1).

**Table 1.** Socio-demographic and lifestyle-related factors associated with LBP and LBP prevalence (n = 207)

Variables		Study sample		LBP point prevalence		LBP 6 months prevalence		LBP 12 months (1 year) prevalence	
		Frequency	Percent	LBP Yes (%)	P value	LBP Yes (%)	P value	LBP Yes (%)	P value
**Gender**	Male	92	44.4	20.7	0.144	38	0.023	52.2	0.003
	Female	115	55.6	29.6		53.9		72.2	
**Age group (years)**	≤ 21	76	36.7	19.7	0.160	47.4	0.942	63.2	0.755
	22-24	108	52.2	26.9		47.2		64.8	
	≥ 25	23	11.1	39.1		43.5		56.5	
**Education level**	1 ^st^ year	31	15	16.1	0.541	35.5	0.263	45.2	0.161
	2 ^nd^ year	31	15	25.8		58.1		77.4	
	3 ^rd^ year	30	14.5	33.3		56.7		70	
	4 ^th^ year	35	16.9	25.7		42.9		65.7	
	Final year	40	19.3	20		37.5		60	
	Internship	40	19.3	32.5		52.5		62.5	
**BMI**	≤ 25	127	61.4	22.8	0.250	39.4	0.007	56.7	0.013
	> 25	80	38.6	30		58.8		73.8	
**Physical activity frequency**	Low	94	45.4	28.7	0.05	55.3	< 0.005	72.3	< 0.005
	Moderate	69	33.3	30.4		52.2		72.5	
	High	44	21.3	11.4		20.5		29.5	
**Smoking habit**	Smoker	27	13	22.2	0.606	55.6	0.099	74.1	0.160
	Ex-smoker	8	3.9	12.5		12.5		37.5	
	Non-smoker	172	83.1	26.7		47.1		62.8	
**Sitting time (hours/day)**	< 6	64	30.9	18.8	0.131	31.3	0.003	45.3	< 0.005
	≥ 6	143	69.1	28.7		53.8		71.3	
**Chair type**	Have back support	158	76.3	20.9	0.005	41.1	0.003	58.9	0.018
	No back support	49	23.7	40.8		65.3		77.6	
**Chair’s back support**	Adjustable	72	34.8	13.9	0.005	27.8	< 0.005	37.5	< 0.005
	Non-adjustable	135	65.2	31.9		57		77	
**Chair’s sitting surface**	Adjustable	92	44.4	16.3	0.006	32.6	< 0.005	46.7	< 0.005
	Non-adjustable	115	55.6	33		58.3		76.5	
**Rest time**	Enough	166	80.2	24.1	0.317	40.4	< 0.005	56	< 0.005
	Not enough	41	19.8	31.7		73.2		92.7	
**Most activity done in a day (by)**	No task for long time	82	39.6	13.4	0.006	24.4	< 0.005	40.2	< 0.005
	Sitting	100	48.3	35		65		83	
	Standing or walking	17	8.2	35.3		52.9		52.9	
	Bending	8	3.9	12.5		37.5		75	

### LBP prevalence and LBP characteristics

The point, 6-month, and 12-month prevalence of LBP was 25.6%, 46.9%, and 63.3%, respectively. Nearly two-thirds (65.9%) of participants with LBP informed that they suffered the first episode of LBP after being admitted in medical, while only 5.5% experienced it during the internship. 35.1% of participants reported that they experienced only one episode of LBP, whereas 35.1% experienced 2–3 episodes, and 29.8% experienced more than three episodes in the previous 12 months. More than half of the respondents (55%) had a short duration (1 – 7 days) of LBP, while 8.4% reported they had LBP every day in the past year. ^27^

As for causes or diagnosis of LBP, the majority (70.8%) reported no diagnosis, therefore had non-specific LBP. More than half of the participants (53.2%) reported no associated leg pain, while 31.2% reported radiated leg pain. Regarding aggravating factors, more than half of the participants (55.2%) reported that LBP worsened when they maintained a static position for a long time followed by bending or twisting (18.8%), lifting any object (8.4%), sudden movement (5.2%), performing repetitive tasks (3.2%) and non-specific (9.1%). Three-fifths of the participants (60.4%) reported their pain relieved without taking any specific treatment while the remaining received different medications in the form of opioid analgesics (22.1%), exercise therapy (9.1%), steroid injection (1.3%), both opioid and exercise therapy (5.2%), and opioid with steroid injection (1.9%) ( Table 2).

**Table 2.** Characteristics of low back pain among Bangladeshi medical students, 2020 (n = 207).

		Males (%)	Females (%)	Total (%)
**Experienced a major episode of LBP for the first time**	As an intern doctor	8.1	3.9	5.5
	As a medical student	66.1	65.7	65.9
	As a college student	17.7	19.6	18.9
	As a school student	8.1	10.8	9.8
**Duration of LBP in last 12 months**	(1 – 7) days	52.1	56.6	55
	(8 – 30) days	20.8	15.7	17.6
	> 30 days	18.8	19.3	19.1
	Everyday	8.3	8.4	8.4
**LBP episodes in last 12 months**	1	37.5	33.7	35.1
	(2 – 3)	33.3	36.1	35.1
	> 3	29.2	30.1	29.8
**Causes of LBP**	No diagnosis or non-specific	71.2	70.5	70.8
	Ligament sprain	3.4	3.2	3.2
	Muscle strain	6.8	9.5	8.4
	Neuropathy	1.7	0	0.6
	Vertebral disc involvement	0	3.2	1.9
	Degeneration	1.7	0	0.6
	Back trauma and fracture	10.2	3.2	5.8
	Others	5.1	10.4	8.4
**LBP associated with leg pain**	Yes	23.7	35.8	31.2
	No	64.4	46.3	53.2
	Maybe	11.9	17.9	15.6
**Aggravating** **factors**	Bending or twisting	23.7	15.8	18.8
	Lifting any object	10.2	7.4	8.4
	Maintaining a position for long time	45.8	61.1	55.2
	Sudden movement	5.1	5.3	5.2
	Performing repetitive tasks	3.4	3.2	3.2
	Non-specific	11.9	7.4	9.1
**Treatment received**	Opioid painkillers	20.3	23.2	22.1
	Steroid injections	1.7	1.1	1.3
	Exercise therapy	13.6	6.3	9.1
	Psychological counselling	0	0	0
	Opioid + exercise	3.4	6.3	5.2
	Opioid + steroid injection	1.7	2.1	1.9
	No treatment	59.3	61.1	60.4

### Relationship between socio-demographic factors and LBP prevalence

Bivariate analysis showed no significant association between LBP and age groups (p > 0.160) or the education level of participants (p > 0.161) regardless of the time of occurrence ( Table 1).

In contrast, the 6-month and 12-month prevalence of LBP was significantly correlated with gender or being overweight. The number of females with LBP was more than the number of males with LBP during the survey (20.7% vs 29.6%, p = 0.144), in the last 6 months (38% vs 53.9%; p = 0.023) and in the last 12 months (52.2% vs 72.2%; p = 0.003). In addition, participants with BMI > 25 kg/m ^2^ reported the presence of LBP more frequently than the participants with BMI ≤ 25 kg/m ^2^ during the survey (22.8% vs 30%), in the past 6 months (39.4% vs 58.8%) and in the last 12 months (56.7% vs 73.8%) ( Table 1).

In the logistic regression analysis, females were 2.3 times more likely to have LBP compared to males (OR: 2.4, 95% CI: 1.3 – 4.2; p = 0.003), and the participants with BMI > 25 kg/m ^2^ were around two times at higher risk of developing LBP than the participants with BMI ≤ 25 kg/m ^2^ (OR: 2.1, 95% CI: 1.2 – 3.9; p = 0.014) in the last 12 months ( Table 3).

**Table 3.** Logistic regression analysis of factors associated with low back pain (n = 207).

Variables		OR (95% CI)
**Gender**	Male	1
	Female	2.4 (1.3 – 4.2) ^*^
**Age group (years)**	≤ 21	1
	22-24	1.1 (0.6 – 1.9) ^*^
	≥ 25	0.8 (0.3 – 1.9) ^*^
**Education level**	1 ^st^ year	1
	2 ^nd^ year	4.2 (1.4 – 12.5) ^*^
	3 ^rd^ year	2.8 (0.9 – 8.1) ^*^
	4 ^th^ year	2.3 (0.9 – 6.3) ^*^
	Final year	1.8 (0.7 – 4.7) ^*^
	Internship	2.0 (0.8 – 5.3) ^*^
**BMI (kg/m** ^**2**^ **)**	≤ 25	1
	> 25	2.1 (1.2 – 3.9) ^*^
**Physical activity frequency**	High	1
	Moderate	6.3 (2.7 – 14.5) ^**^
	Low	6.2 (2.8 – 13.8) ^**^
**Smoking habit**	Non-smoker	1
	Ex-smoker	0.4 (0.1 – 1.5) ^*^
	Smoker	1.7 (0.7 – 4.2) ^*^
**Sitting time (hours/day)**	< 6	1
	≥ 6	3.0 (1.6 – 5.5) ^**^
**Chair type**	Have back support	1
	No back support	2.4 (1.2 – 5.1) ^*^
**Chair's back support**	Adjustable	1
	Non-adjustable	5.6 (2.9 – 10.4) ^**^
**Chair's sitting surface**	Adjustable	1
	Non-adjustable	3.7 (2.1 – 6.7) ^**^
**Rest time**	Enough	1
	Not enough	9.9 (2.9 – 33.5) ^**^
**Most activity done in a day (by)**	No task for long time	1
	Sitting	7.3 (3.7 – 14.4) ^**^
	Standing or walking	1.7 (0.6 – 4.8) ^*^
	Bending	4.4 (0.8 – 23.4) ^*^

* Indicates p – value < 0.05.

** Indicates p – value < 0.005.

### Relationship between lifestyle factors and LBP prevalence

According to the bivariate analysis, the factors that significantly contributed to LBP occurrence in the last 6 months and 12 months were frequency of physical activity, total sitting time per day, availability of rest time, and type of activity mostly done in a day. However, the point prevalence of LBP was significantly correlated with only physical activity and the type of activity mostly done in a day. Results demonstrated that the respondents who performed a high frequency of physical activity, those who spent < 6 hours per day by sitting, those who had enough rest time, and those who did not perform any specific task for a long time had the least prevalence of LBP in all time points compared to their counterparts ( Table 1).

Results of logistic regression analysis showed that the participants who performed moderate and low frequency of physical activity were 6.3 times (OR: 6.3, 95% CI: 2.7 – 14.5; p < 0.005), and 6.2 times (OR: 6.2, 95% CI: 2.8 – 13.7, p < 0.005) more likely to develop LBP in last 12 months than the participants who performed a high frequency of physical activity, respectively. Moreover, the odds of LBP were 1.7 times higher among smokers than non-smokers (OR: 1.7, 95% CI: 0.7 – 4.2; p = 0.259), more than three times higher among participants who spent ≥ 6 hours in sitting than those spent < 6 hours (OR: 3.0, 95% CI: 1.6 – 5.5; p < 0.005), and almost 10 times higher among the subjects who had insufficient rest time than those who had enough rest time (OR: 9.9, 95% CI: 2.9 – 33.5; p < 0.005). In addition, the participants who did most of the activity in a day by sitting, standing, or walking, and bending were about 7.3 times (OR: 7.3, 95% CI: 3.7 – 14.4; p < 0.005), 1.7 times (OR: 1.7, 95% CI: 0.6 – 4.8; p = 0.338) and 4.5 times (OR: 4.5, 95% CI: 0.8 – 23.4; p = 0.078) more likely to suffer from LBP in last year compared to the participants who did not perform any activity in a specific position for a long time, respectively ( Table 3).

### Relationship between participants’ chair type and LBP prevalence

Bivariate analysis revealed that the prevalence of LBP, regardless of the time of occurrence, was significantly correlated with the presence of back support, adjustable back support, and adjustable sitting surface of participants’ chairs. Results showed that the participants who had chairs with back support, adjustable back support, and adjustable sitting surface had a lower LBP prevalence than their counterparts ( Table 1).

Further analysis showed that the 12-month prevalence of LBP was about 2.5 times higher among participants who used chairs without back support (OR: 2.4, 95% CI: 1.2 – 5.1; p = 0.020), nearly 5.6 times higher among participants who used chairs without adjustable back support (OR: 5.6, 95% CI: 2.9 – 10.4; p < 0.005), and almost 3.7 times higher among participants who used chairs without adjustable sitting surface (OR: 3.7, 95% CI: 2.0 – 6.7; p < 0.005) compared to their respective reference group ( Table 3).

## Discussion

### LBP prevalence

The results of our study indicated that almost half (46.9%) and two-thirds (63.3%) of the participants experienced LBP in the past 6 months and 12 months, respectively, while 25.6% reported LBP at the time of the survey. Compared to this study, 12-month prevalence of LBP was lower among the medical students in Pakistan (38.6%), ^19^ China (40.1%), ^20^ the US (42.8%), ^21^ Malaysia (46.1%), ^22^ India (47.5%), ^10^ Austria (53.4%), ^14^ Serbia (59.5%), ^5^ Brazil (59.9%), ^23^ Saudi Arabia (61.4%), ^20^ and was higher among the medical students in Turkey (96.4%). ^34^ The discrepancy in the LBP prevalence could be from some factors, including the variation of faculty year of study, academic curriculum, methodological heterogenicity, mode of data collection, cross-cultural factors, and subjective perception of pain. ^35^^,^^36^

### Socio-demographic factors and LBP prevalence

The findings of the study showed that sex and weight are two socio-demographic factors that were associated with 12-month prevalence of LBP among Bangladeshi medical students. Results showed that females had a significantly higher prevalence of LBP than males, which was consistent with several studies. ^5^^,^^12^^,^^30^^,^^37^^,^^38^ Males are structurally, anatomically, and physiologically different from females, and researchers asserted that females have lower pain thresholds and higher sensitivity to pain than males. ^39^^,^^40^ For these reasons, females are more likely to report LBP than males. Although, some studies did not reveal any significant association between LBP prevalence and gender. ^10^^,^^20^^,^^22^^,^^38^^,^^41^ In addition, participants with BMI > 25 kg/m ^2^ had a higher prevalence of LBP than the participants with BMI ≤ 25 kg/m ^2^, which is comparable with the findings of a meta-analysis by Shiri *et al.* ^42^ and a study by Webb *et al.* ^43^ Researchers showed that as weight increases, it creates higher pressure on the intervertebral disc and other spine structures, consequently triggers pain. ^44^ However, few studies did not find any association between weight and LBP prevalence. ^11^^,^^34^^,^^38^^,^^41^

In contrast, several studies have stated that the prevalence of LBP increases with age, ^11^^,^^45^ although some studies revealed that the prevalence of LBP was higher among younger nurses than older nurses. ^46^^–^^48^ Contrary to these findings, our study demonstrated no significant relationship between age and prevalence of LBP, which is comparable with several studies. ^41^^,^^49^ Moreover, several studies demonstrated a significant correlation between the year of study with MSP, including LBP among medical students. ^13^^,^^22^^,^^23^^,^^38^ However, the results of our study indicated no association between the year of the study and LBP prevalence, although the second-year students had a higher prevalence of LBP than others. The reason could be that the second-year students need to appear for their first professional exam, and at that time, different factors, including more study hours, stress, and psychological imbalance, could evoke LBP. In contrast, few studies did not find any association between MSP prevalence, including LBP, and study year among medical students. ^19^^,^^24^^,^^41^^,^^49^

### Lifestyle factors and LBP prevalence

The results of our study revealed that physical activity patterns and total sitting hours were significantly associated with the 12-month prevalence of LBP among the participants. Prolonged sitting is a risk factor of LBP ^50^^,^^51^ as it increases spinal compression load and dysfunction of paraspinal muscles. ^52^^,^^53^ Nyland and Grimmer ^51^ affirmed that ‘a sitting and looking down position’ was a potential risk factor of LBP, and studies demonstrated a positive correlation between staying in a sitting position for a long time and LBP. ^34^^,^^41^^,^^54^ Our study revealed that participants who spent ≥ 6 hours sitting had a significantly higher prevalence of LBP than participants who spent < 6 hours sitting. Conversely, Hartvigsen *et al.*, ^55^ Spyropoulos *et al.*, ^30^ and Tavares *et al.*, ^49^ reported no association between sitting time and LBP prevalence. Moreover, medical students generally remain busy with their classes and hospital visits, making their life sedentary. A study on medical students of Delhi showed that only one-third of the medical students performed the recommended amount of physical activity. ^56^ Physical exercise or regular sports practice are encouraged in different studies as it helps to minimize the rate of LBP prevalence and is effective for primary and secondary prevention of LBP. ^57^ The findings of our study demonstrated a significant relationship between the prevalence of LBP and frequency of physical activity, which was supported by previous studies. ^5^^,^^38^ Moreover, The American College of Sports Medicine recommended that to promote and maintain health, physical activity should be performed for at least 30 minutes at moderate intensity with a minimum frequency of 5 days/week. ^58^^,^^59^ The findings of our study were in line with this recommendation as the participants who performed high frequency (≥ five days/week) of physical activity had a significantly lower prevalence of LBP than those who performed low or moderate frequency (< five days/week) of physical activity. However, several studies did not reveal any significant association between physical activity and LBP prevalence among medical students. ^10^^,^^23^^,^^30^^,^^34^^,^^60^

Conversely, our study revealed no association between smoking habit and LBP prevalence at any time point, in accordance with other studies. ^13^^,^^19^^,^^20^^,^^24^^,^^61^ While Shiri *et al.* ^62^ found the correlation between smoking habits and LBP prevalence in their meta-analysis, and they reported that smokers and ex-smokers had a higher prevalence of LBP than non-smokers. Other studies also revealed that medical students who smoked were more likely to suffer LBP. ^41^^,^^60^ This discrepancy could be for the low number of smokers as it can be inferred that studies with less than 10% prevalence of smoking did not find any association between smoking habits and LBP prevalence. Moreover, research showed a positive relationship between the risk of LBP and smoking dose. ^63^ Our study did not assess the intensity of smoking and the duration of exposure to the habit.

### LBP characteristics

We found that in the majority of cases (64.9%), LBP was recurrent, and more than half (55%) of the participants had a short annual duration (1–7 days) of LBP which was consistent with previous studies on Greek public office workers, ^30^ and nursing students and graduate nurses. ^29^ That indicated the high chance of LBP recurrence and chronicity in the future. In addition, most of the participants (53.2%) had localized LBP, and 31.2% reported radiated leg pain, which was consistent with several studies. ^64^^,^^65^

El-soud *et al.* ^65^ found that LBP was non-specific in most cases. In agreement with that result, our study also revealed that 70.8% of the subjects had non-specific LBP. Regarding treatment, the majority (60.4%) of participants reported they did not seek any medication for their symptoms, which is comparable with the findings of studies from Wong *et al.* (65.9%), ^64^ Hafeez *et al.* (64.5%) ^60^ and Falavigna *et al.* (67.3%). ^23^ Moreover, maintaining a specific posture, including standing and sitting, for a long time was the most cited (55.2%) aggravating factor for LBP in our study. In agreement with our finding, previous studies ^66^ also reported LBP mostly worsened after prolonged standing/sitting.

Hestbaek *et al.* ^67^ claimed that the lifetime prevalence of LBP increases markedly between 12 and 22 years of age. Our study came to the same conclusion as we found that 28.7% and 65.7% of participants had LBP before commencing their medical studies and during medical studies, respectively; and more than half (50.4%) of the participants who had LBP in the last 12 months aged ≤ 22 years. That indicated the importance of implementing the LBP prevention strategies before or at the beginning of the medical course.

### Type of chair and LBP prevalence

Individuals suffering from different musculoskeletal pains due to prolonged sitting are recommended to use ergonomically sound chairs as the chair directly influences body alignment or posture. ^68^^,^^69^ Researchers concluded that scarcity of knowledge, understanding, or application of ergonomics’ basic principles and rules could lead to LBP. ^70^ Moreover, it is vital to adjust the height of the sitting surface of the chair to meet individual biomechanical requirements so that they can use the desk with ease without aggravating the spine. ^71^^,^^72^ Our results showed that the participants who used chairs with back support, adjustable back support, and the adjustable sitting surface had a significantly lower prevalence of LBP compared to their counterparts at all time points. However, previous studies did not show any significant association between LBP prevalence and using a chair with back support ^30^^,^^60^ or using a chair with an adjustable sitting surface. ^30^ Whereas Makhsous *et al.* ^73^ and Spyropoulos *et al.* ^30^ demonstrated that LBP prevalence could be lowered using chairs with back support.

### Strengths and limitations

This study was the first study that estimated the prevalence of LBP among Bangladeshi medical students, and the response rate of the study was satisfactory. However, our study has some limitations that should be acknowledged. First, as we included students from only one medical college, the outcomes may not fully represent the situation for all medical students in Bangladesh. Second, the study outcomes relied solely on the self-administrated questionnaire, and we did not perform any medical tests to confirm the presence of LBP. Therefore, information bias and subject bias cannot be ruled out. Moreover, the difficulty in recalling arises the possibility of over-or underreporting of LBP as participants reported the presence of LBP in the last one year, which entirely depended on the participants’ memory. Finally, as it was a cross-sectional study, the exposure to risk factors and outcomes were evaluated concurrently. Hence, we showed only the relationship but could not establish any evidence of the causal association between exposure and LBP occurrence.

## Conclusion

The overall results of our study demonstrated the high prevalence of LBP among Bangladeshi medical students and indicated the necessity of formulating and implementing comprehensive preventive strategies. The majority of the risk factors are modifiable. Hence, initiatives can be taken to inspire them to avoid those risk factors, which could improve medical students’ and future doctors’ overall health and quality of life. Students should be encouraged to perform the recommended amount of physical activity by providing education and facilities and reserving a couple of hours exclusively for exercise and sports activities. Moreover, education on ergonomics and providing sound ergonomic chairs to the students could help minimize LBP prevalence.

Future studies should be undertaken with a larger sample size by including students from more than one medical college. Epidemiological longitudinal studies should be conducted to confirm the association of risk factors with LBP.

## Data availability

### Underlying data

Mendeley Data: Low back pain and associated risk factors among medical students in Bangladesh: A cross-sectional study. https://doi.org/10.17632/mfky2jttwp.3. ^27^

The project contains the following underlying data:

• Raw dataset.xlsx

### Extended data

Mendeley Data: Low back pain and associated risk factors among medical students in Bangladesh: A cross-sectional study. https://doi.org/10.17632/mfky2jttwp.3. ^27^

The project contains the following extended data:

• Course Evaluation – Google Forms.pdf
• STROBE Checklist.doc

Data are available under the terms of the Creative Commons Attribution 4.0 International license (CC-BY 4.0).
