# Supplementary material for: Low back pain and associated risk factors among medical students in Bangladesh: a cross-sectional study
Source: F1000Res. 2022 Jul 25;10:698. Originally published 2021 Jul 30. [Version 3] doi: 10.12688/f1000research.55151.3 (PMC9360907; doi:10.12688/f1000research.55151.3)
Supplement: Peer review history

# Peer review: Reviewer response for version 3

**DOI:** 10.5256/f1000research.136001.r145290

**Published:** 2022-08-08

**Authors:** Matthew Chiwaridzo

**Affiliations:** 1 Faculty of Medicine and Health Sciences, Department of Primary Health Care Sciences, University of Zimbabwe, Harare, Zimbabwe

I have no further comments.

Is the work clearly and accurately presented and does it cite the current literature?

Partly

If applicable, is the statistical analysis and its interpretation appropriate?

Yes

Are all the source data underlying the results available to ensure full reproducibility?

Yes

Is the study design appropriate and is the work technically sound?

Yes

Are the conclusions drawn adequately supported by the results?

Yes

Are sufficient details of methods and analysis provided to allow replication by others?

Yes

Reviewer Expertise:

Low back pain

I confirm that I have read this submission and believe that I have an appropriate level of expertise to confirm that it is of an acceptable scientific standard.

# Peer review: Reviewer response for version 2

**DOI:** 10.5256/f1000research.120892.r122789

**Published:** 2022-03-08

**Authors:** Matthew Chiwaridzo

**Affiliations:** 1 Faculty of Medicine and Health Sciences, Department of Primary Health Care Sciences, University of Zimbabwe, Harare, Zimbabwe

Thank you for the opportunity to review this manuscript again.

 Let me commend the authors for an interesting paper addressing a significantly important health issue in Bangladesh about the prevalence of low back pain and associated risk factors among medical students.

 I have minor comments only.  

**General comment:**

1. "These studies showed a high prevalence of LBP. However, regarding the associated risk factors, the findings were inconsistent"
  Kindly expand by adding a statement or two, providing the evidence on the inconsistencies, referencing current studies validating your point above. 
2. "No formal ethical approval was required from the institution; hence, no approval number was provided. The institution requires formal ethical approval for clinical trials involving human participants."
  Check with the journal requirements or the handling editor for this part. My understanding is that primary research with human participants requires ethical approval prior to conduction. 
3. "Because of the possibility of sample loss, the final sample size was determined as 270”:
  Provide the minimum sample calculated first from estimated prevalence figures, precision effect, and confidence interval parameters and how the maximum sample of 270 was arrived at. This helps with the reproduction of the study in other settings. 

No further comments.

Is the work clearly and accurately presented and does it cite the current literature?

Partly

If applicable, is the statistical analysis and its interpretation appropriate?

Yes

Are all the source data underlying the results available to ensure full reproducibility?

Yes

Is the study design appropriate and is the work technically sound?

Yes

Are the conclusions drawn adequately supported by the results?

Yes

Are sufficient details of methods and analysis provided to allow replication by others?

Yes

Reviewer Expertise:

low back pain

I confirm that I have read this submission and believe that I have an appropriate level of expertise to confirm that it is of an acceptable scientific standard, however I have significant reservations, as outlined above.

# Response

**Published:** 2022-03-09

**Authors:** Shabbir Ahmed Sany

**Affiliations:** Faridpur Medical College, Bangladesh

Thank you for your valuable time to review this manuscript again. Also, we are grateful to you for your comments which will increase the quality of the manuscript. We want to respond to your comments first before submitting the revised version of the manuscript. We will be happy to submit the revised manuscript (if required) if our responses do not suffice to get approval.

*1. Kindly expand by adding a statement or two, providing the evidence on the inconsistencies, referencing current studies validating your point above.*

**Response**: Thank you for your comment. However, inconsistencies in findings were described in detail in the ‘Discussion’ section. As the inconsistencies were observed in the case of different risk factors, it will be difficult to refer to every study. Therefore, we did not include any statement regarding this in the introduction section.

*2. Check with the journal requirements or the handling editor for this part. My understanding is that primary research with human participants requires ethical approval prior to conduction*.

**Response**: Thank you for pointing this out. Permission was taken from the ERC of Faridpur Medical College. However, this institution's ERC usually does not assign any approval number unless the study is a clinical trial. As our study was questionnaire-based, and there was no human participation in the lab, permission from ERC sufficed the ethical requirement.

*3. Provide the minimum sample calculated first from estimated prevalence figures, precision effect, and confidence interval parameters and how the maximum sample of 270 was arrived at. This helps with the reproduction of the study in other settings.*

**Response**: Thank you for your comment. In the method section (under subheading ‘Study settings and population’), we stated that the study’s sample size was calculated as 251 using OpenEpi version 3.1, assuming 47.5% as the estimated prevalence rate at a 95% confidence level with 5% precision.

# Peer review: Reviewer response for version 2

**DOI:** 10.5256/f1000research.120892.r122790

**Published:** 2022-02-23

**Authors:** Mohammad Mostafa Zaman

**Affiliations:** 1 Non-Communicable Disease Unit, World Health Organization Country Office for Bangladesh, Dhaka, Bangladesh

The authors prefer using one-year prevalence data for the logistic regression although they have point prevalence and 6-month prevalence data. The recall bias will be less if the recall duration is shorter. Therefore it would be good to have factors associated with the point-and 6-month prevalence. I suggest adding, at least, an in-text description to have the results of models for point prevalence and a 6-month recall. Necessary comments can be given in the Discussion section. This will have a high potential for citation because future studies may use different recall periods.

Is the work clearly and accurately presented and does it cite the current literature?

Partly

If applicable, is the statistical analysis and its interpretation appropriate?

Yes

Are all the source data underlying the results available to ensure full reproducibility?

Yes

Is the study design appropriate and is the work technically sound?

Yes

Are the conclusions drawn adequately supported by the results?

Yes

Are sufficient details of methods and analysis provided to allow replication by others?

Yes

Reviewer Expertise:

Epidemiology of NCD risk factors, rheumatic disorders

I confirm that I have read this submission and believe that I have an appropriate level of expertise to confirm that it is of an acceptable scientific standard.

# Response

**Published:** 2022-07-07

**Authors:** Shabbir Ahmed Sany

**Affiliations:** Faridpur Medical College, Bangladesh

The authors prefer using one-year prevalence data for the logistic regression although they have point prevalence and 6-month prevalence data. The recall bias will be less if the recall duration is shorter. Therefore, it would be good to have factors associated with the point-and 6-month prevalence. I suggest adding, at least, an in-text description to have the results of models for point prevalence and a 6-month recall. Necessary comments can be given in the Discussion section. This will have a high potential for citation because future studies may use different recall periods.

**Response**: Thank you for your comment and valuable suggestion. However, it needs further analysis and description of data if we want to report the factors related to the point and 6-month recall period. That will make the manuscript very long.

# Peer review: Reviewer response for version 1

**DOI:** 10.5256/f1000research.58703.r95514

**Published:** 2021-10-18

**Authors:** Matthew Chiwaridzo

**Affiliations:** 1 Faculty of Medicine and Health Sciences, Department of Primary Health Care Sciences, University of Zimbabwe, Harare, Zimbabwe

Thank you for the opportunity to review this interesting manuscript.

 Although the study addresses an interesting research question for Bangladesh, and perhaps other countries, there is more detail that I would like to see in the introduction and methods sections, as well as integration of a few additional references and concepts throughout the manuscript. As such, this manuscript will require minor revisions to meet the criteria for indexing.

 As the research design is observational, the authors are commended for applying the STROBE guidelines (available via the equator network at http://www.equator-network.org/reporting-guidelines/).

**Introduction**

- This paper needs to strongly emphasise its unique contribution to the literature base in the context of what other authors have already researched and reported with regards to low back pain among medical students. LBP among medical students or generally university students is a well-researched area globally with studies from India, Pakistan, China, USA, Brazil, South Africa, Zimbabwe, Austria, and Malaysia.
- The theoretical perspective to the problem is narrow in light of the many studies that have been conducted to answer the research question the authors are posing. Admittedly, the authors pointed many studies reporting a high prevalence of LBP among medical students from elsewhere.
- The authors should briefly describe the results of some of the referenced studies in the background so that we can understand the theoretical background of this study.
  - What was the prevalence of LBP among medical students from other studies?
  - Which type of LBP was investigated and how was the investigation carried out?
  - Where were these studies carried out and how do the results of these few studies mentioned in the background apply or not apply to the Bangladesh medical students’ situation?
  - What factors affect the extrapolation of results?
  - In what way are medical students from Bangladesh different from medical students from other countries like India, Pakistan for example?
- The authors should also highlight the contextual background underpinning the conduction of the study in Bangladesh, Faridpur Medical College.
  - What prompted the authors to want to do this study?
  - What local problem justifies this study?
  - What was observed by the authors among medical students in Bangladesh strongly justifying the need for this study?
- I commend the authors for giving us a brief narration of the MBBS programme but more can be added to show how exposed the students are to LBP and what exposes them. How is that exposure so unique to the Bangladeshis?
- The authors are reminded that a statement like this “…no study has been conducted to evaluate the prevalence of LBP among Bangladeshi medical students” is not a sound and adequate justification. What is the problem now that has prompted an investigation into the prevalence of LBP and associated factors among medical students in Bangladesh particularly in the setting the study was conducted? Identification of the specific problem in Bangladesh makes the study uniquely different from similar studies conducted elsewhere.
- Provide a hint on the significance of this study in your context.

**Methods**

- Clearly provide details of the psychometric properties of the LBP questionnaire used in the study. How was the questionnaire tested for reliability and logical validity against the set objectives?
- Kindly reference your operational variables such as point prevalence.
- Not clear whether the authors are addressing non-specific or specific LBP, acute vs chronic until the results section.
- Reference 6 is the same as reference 22. Kindly check and address.
- For Sample size calculation: Which recall period (point, 6-month or 12-month) was used for the estimation of the sample size? Kindly provide a justification for adopting a prevalence figure from the following study by Aggarwal *et al*., 2017 ^1^ to inform your sample size calculation.
- “Because of the possibility of sample loss, the final sample size was determined as 270”: Be clear how that was arrived at.
- Did the authors check the continuous variables for normality first, and if so what tests were used?
- Clarify the Inclusion and exclusion criteria of the participants.
- The Ethical approval number from the “institute” should be provided.

**Results**

- Your final sample size was less than the calculated minimum sample size?
- The use of a flow chart is commendable.
- Statistical analysis is appropriate and well explained.

**Discussion and conclusion**

- May I suggest removing sub-headings in the discussion section for clarity.
- The first paragraph of the discussion should summarise the main findings from the study addressing the main research question.
- Discuss briefly your findings on the association between age and LBP as they contrasted many findings from the literature.
- Study limitations are well explained.
- “The overall results of our study demonstrated the high prevalence of LBP among Bangladeshi medical students and indicated the necessity of formulating and implementing comprehensive preventive strategies”: May I suggest you expand briefly on the possible preventative strategies based on your study findings. This is the most important part of this study. What can be done to curtail the problem of LBP among medical students given contextual information and study results? 

**References **

- Lack of consistency in referencing style in the reference list.
- References 6 and 22 duplicated.
- Add URL links for internet-derived references and dates accessed.

Is the work clearly and accurately presented and does it cite the current literature?

Partly

If applicable, is the statistical analysis and its interpretation appropriate?

Yes

Are all the source data underlying the results available to ensure full reproducibility?

Yes

Is the study design appropriate and is the work technically sound?

Yes

Are the conclusions drawn adequately supported by the results?

Yes

Are sufficient details of methods and analysis provided to allow replication by others?

Yes

Reviewer Expertise:

NA

I confirm that I have read this submission and believe that I have an appropriate level of expertise to confirm that it is of an acceptable scientific standard, however I have significant reservations, as outlined above.

## References

[1] : Low back pain and associated risk factors among undergraduate students of a medical college in Delhi. *Educ Health (Abingdon)* .26(2) : 10.4103/1357-6283.120702 103-8 10.4103/1357-6283.120702 24200731

# Response

**Published:** 2022-01-31

**Authors:** Shabbir Ahmed Sany

**Affiliations:** Faridpur Medical College, Bangladesh

Thank you for giving us the opportunity to submit a revised draft of our manuscript. We would like to thank you for your insightful comments on the paper, as these comments led us to improve the quality of the manuscript. We took into consideration all comments and concerns in the paper. Detailed responses are given below:

**Introduction**:

- Thank you for your valuable suggestion. We have made some changes as you suggested, especially we described the necessity of conducting the current study. Although several studies have been conducted globally to determine the prevalence of LBP, the results were inconsistent. Particularly regarding the risk factors, the results were contradictory as the socio-demographic factors of participants, study design, and exposure to risk factors were not the same. Therefore, this study was conducted to estimate the prevalence of LBP among medical students of Bangladesh. The results of this study will help to better know about the risk factors and their impact. We can also compare the results with other studies which will help to examine the validity of evidence and take initiatives to prevent LBP.
- We have also included the studies that determined LBP prevalence among medical students of different countries.

**Methods**:

- The questionnaire was piloted on 15 students before administration in the study to confirm the validity and reliability.
- The reference for the definition of operational variables was included.
- Determining the acute vs. chronic, non-specific vs. specific LBP was not the study's primary aim. We just showed the percentage of participants who had non-specific and specific LBP.
- Test of Normality for continuous variables was not performed as we categorized the continuous variables for analysis.
- We included the inclusion and exclusion criteria briefly as you suggested.
- No formal ethical approval was required from the institution; hence, no approval number was provided. The intuition requires formal ethical approval for clinical trials involving human participants.
- We followed the STROBE guidelines. The checklist of the STROBE guidelines can be found at the Mendeley data repository ^1^. 

**Results**:

- A flow chart was included as 'Figure 1', which demonstrated the different phases of the study.
- Two hundred twenty-three participants responded, and then 16 were excluded as they did not respond fully. Therefore, a total of 207 respondents were included for analysis.

**Discussion and conclusion**:

- Changes were made where you suggested. Some sub-headings were removed for clarity. The association between age and prevalence of LBP was discussed under the subheading titled 'Socio-demographic factors and LBP prevalence.'
- The preventive measure was discussed briefly. It would have been better to expand it. However, in the case of our study, it seems slightly out of scope. Future studies involving medical students with more than one medical college are warranted for better evidence and giving the appropriate recommendation for implementing preventive measures.

**References**:

- The correction was done as you suggested. The reference style was autogenerated by using Mendeley software. Later it was published following the journal’s formatting style.

1. Sany, Shabbir Ahmed; Tanjim, Taukir; Hossain, Md Ikbal (2021), “Low back pain and associated risk factors among medical students in Bangladesh: A cross-sectional study”, Mendeley Data, V4, doi: 10.17632/mfky2jttwp.4.

# Peer review: Reviewer response for version 1

**DOI:** 10.5256/f1000research.58703.r93460

**Published:** 2021-09-27

**Authors:** Mohammad Mostafa Zaman

**Affiliations:** 1 Non-Communicable Disease Unit, World Health Organization Country Office for Bangladesh, Dhaka, Bangladesh

LBP is a common problem in Bangladesh and elsewhere. Data for medical students are lacking. This survey has been done among students of a medical college in Bangladesh (Faridpur Medical College). The manuscript will contribute to LBP prevention measures in the medical profession. However, there are points of concern:

**1. Abstract**

- The objective was to evaluate the prevalence. The authors have not done any evaluation. "Evaluated" could be replaced by "determined".
- 70.8% were undiagnosed is not correct; the authors have not diagnosed LBP patients that were not diagnosed before. 

**2. Introduction**

- The literature review is incomplete. National survey findings have been published in 2020, I do not see it in the review. For example, Zahid-Al-Quadir *et al.* ^1^ reported the burden of LBP in the general population, and the introduction could build on this and the discussion could also benefit from adding this.

**3. Methods**

- Sample size calculation is based on an Indian study, which is very high. Prevalence in Bangladesh is much lower. There was another COPCORD study that reported LBP among other rheumatic disorders.
- Logistic regression analysis was done for a 12-month recall period, which has a potential of recall bias; this could be done for point prevalence.
- Ethical clearance has been obtained from the institute. Which institute?

**4. Results**

- The results described have many digits after the decimal point; these could be up to one decimal point. No message will be lost by removing extra decimal points.
- Dividing a small number of students (207) into so many age and educational groups is meaningless. There are hardly any points of implications by age and educational years, the differences in education and age are obviously very narrow. The message of the study could be generated for one group only. At best, the results could be presented for male and female, and students and interns.
- Providing both CIs and P-values is redundant information. The authors could put esoteric marks against significant CIs (Table 3).

**5. Discussion**

- The discussion is quite long. There are so many subheadings used because authors have discussed all variables under study. They could highlight the main points of the message that the study transmits to the readers. For example, years cannot be changed or intervened separately but it has a lengthy description.
- The last paragraph under the Conclusion is a repetition of the limitations mentioned above; this could be deleted.

**6. Reference**

- Reference 72 is a duplication of reference 24.

Is the work clearly and accurately presented and does it cite the current literature?

Partly

If applicable, is the statistical analysis and its interpretation appropriate?

Yes

Are all the source data underlying the results available to ensure full reproducibility?

Yes

Is the study design appropriate and is the work technically sound?

Yes

Are the conclusions drawn adequately supported by the results?

Yes

Are sufficient details of methods and analysis provided to allow replication by others?

Yes

Reviewer Expertise:

Epidemiology of NCD risk factors, rheumatic disorders

I confirm that I have read this submission and believe that I have an appropriate level of expertise to confirm that it is of an acceptable scientific standard, however I have significant reservations, as outlined above.

## References

[1] : Prevalence of musculoskeletal conditions and related disabilities in Bangladeshi adults: a cross-sectional national survey. *BMC Rheumatol* .2020;4(1) : 10.1186/s41927-020-00169-w 69 10.1186/s41927-020-00169-w 33323124PMC7739446

# Response

**Published:** 2022-01-31

**Authors:** Shabbir Ahmed Sany

**Affiliations:** Faridpur Medical College, Bangladesh

Thank you for giving us the opportunity to submit a revised draft of our manuscript. We appreciate the time and effort you have dedicated to providing your valuable feedback on the manuscript. We are grateful to you for your insightful comments on our paper. We have been able to incorporate changes to reflect most of your suggestions. Here is a point-by-point response to the reviewers' comments and concerns.

**Abstract**:

- *The objective was to evaluate the prevalence. The authors have not done any evaluation. "Evaluated" could be replaced by "determined".*

Response: Thank you for your suggestion. We have replaced the word 'evaluated' with 'determined.'

- *70.8% were undiagnosed is not correct; the authors have not diagnosed LBP patients that were not diagnosed before.*

Response: We agree with this comment. 70.8% were non-specific LBP rather undiagnosed. We have corrected it.

**Introduction**:

- *The literature review is incomplete. National survey findings have been published in 2020, I do not see it in the review. For example, Zahid-Al-Quadir et al.* *^1^* *reported the burden of LBP in the general population, and the introduction could build on this and the discussion could also benefit from adding this*.

Response: Thank you for your suggestion. We have made the changes as you suggested.

**Methods**:

- *Sample size calculation is based on an Indian study, which is very high. Prevalence in Bangladesh is much lower. There was another COPCORD study that reported LBP among other rheumatic disorders*.

Response: Thank you for pointing this out. However, the prevalence of LBP is relatively higher among the people involved with health professions. Physiotherapists in Bangladesh, for instance, had a 60.8% prevalence of LBP, which is higher than other professions [1]. Moreover, our study was the first study that determined LBP prevalence among Bangladeshi medical students. Therefore, we calculated the sample size based on an Indian study as the Bangladeshi medical students have relatively similar curriculum, clinical class exposure, study load, and social and cultural demographics as Indian medical students.

- *Logistic regression analysis was done for a 12-month recall period, which has a potential of recall bias; this could be done for point prevalence.*

Response: You have raised an important point here. However, determining risk factors was one of the study's primary objectives; we opted to do logistic regression analysis for 1-year prevalence instead of point prevalence despite the potential of recall bias. Point prevalence could be too short a period to conclude the effect of exposure of any particular risk factor.

- *Ethical clearance has been obtained from the institute. Which institute?*

Response: Thank you for pointing this out. We have corrected it. The ethical Permission was taken from the Ethical Review Committee (ERC) of Faridpur Medical College.

**Results**:

- *The results described have many digits after the decimal point; these could be up to one decimal point. No message will be lost by removing extra decimal points.*

Response: Agree. We have incorporated your suggestion throughout the manuscript.

- *Dividing a small number of students (207) into so many age and educational groups is meaningless. There are hardly any points of implications by age and educational years, the differences in education and age are obviously very narrow. The message of the study could be generated for one group only. At best, the results could be presented for male and female, and students and interns.*

Response: Thank you for your comment. However, previous studies showed a significant association between medical students' age and education years and LBP prevalence. For this reason, we wanted to observe it among Bangladeshi medical students, and we divided the students into three age groups and academic years. We found that the 2 ^nd^ year medical students had a higher prevalence of LBP, although the difference was not significant.

- *Providing both CIs and P-values is redundant information. The authors could put esoteric marks against significant CIs (Table 3)*.

Response: Thank you for pointing this out. We have modified Table 3 as you suggested.

**Discussion**:

- *The discussion is quite long. There are so many subheadings used because authors have discussed all variables under study. They could highlight the main points of the message that the study transmits to the readers. For example, years cannot be changed or intervened separately but it has a lengthy description.*

Response: Thank you for your suggestion. We have made the changes as you suggested.

- *The last paragraph under the Conclusion is a repetition of the limitations mentioned above; this could be deleted*.

Response: We have deleted the line as you suggested.

**Reference**:

- Reference 72 is a duplication of reference 24.

Response: Thank you for pointing this out. We have corrected it.

 [1]      R. Mondal, R. C. Sarker, S. Akter, P. C. Banik, and S. K. Baroi, “Prevalence of low back pain and its associated factors among physiotherapists in Dhaka city of Bangladesh in 2016,” *J. Occup. Heal. Epidemiol.*, vol. 7, no. 2, pp. 70–74, 2018.
